# Sodium Danshensu promotes diabetic wound healing by targeting the EGFR-mediated PI3K-AKT pathway: a combined network pharmacology, machine learning, and *in vitro* approach

**DOI:** 10.1039/d5ra09417h

**Published:** 2026-03-10

**Authors:** Peng Ning, Fan Yang, Hongyi Cao, Bo Zhou

**Affiliations:** a Department of Endocrinology, The First Affiliated Hospital of Chongqing Medical University No. 1 Youyi Road, Yuzhong District Chongqing 400016 China zhoubo915@126.com; b Department of Endocrine and Metabolism, Chengdu Fifth People's Hospital (The Second Clinical Medical College, Affiliated Fifth People's Hospital of Chengdu University of Traditional Chinese Medicine), Geriatric Diseases Institute of Chengdu/Cancer Prevention and Treatment Institute of Chengdu Chengdu China

## Abstract

Sodium Danshensu (SDSS) shows potential in treating diabetic wounds (DWs) owing to its antioxidant, anti-inflammatory, and pro-angiogenesis effects. The specific pharmacological mechanisms of SDSS in achieving the above effects were evaluated. The potential targets of SDSS and DWs were obtained from online databases. Interaction networks were constructed using network pharmacology, and Gene Ontology and Kyoto Encyclopedia of Genes and Genomes (KEGG) pathway enrichment analyses were performed. Combined with machine learning, the biological targets were evaluated and prioritized. A high-glucose-induced human umbilical vein endothelial cell (HUVEC) model was established for *in vitro* studies. A total of 126 shared targets of SDSS and DWs were selected, and the core targets included EGFR, CASP3, SRC, ESR1, JUN, NFKB1, IGF1R, ESR2, AR, and PPARG. KEGG enrichment analysis revealed significant enrichment of the PI3K-AKT signaling pathway (*P* < 0.05). Machine learning indicated EGFR as a key target of SDSS in treating DWs. Findings from molecular docking and molecular dynamics simulation confirmed the stable combination of SDSS and EGFR. *In vitro* experiments indicated that SDSS may activate the PI3K-AKT pathway *via* EGFR targets, improve the mobility of high-glucose-induced HUVECs, and increase lumen formation (branch number). It promoted catalase production and inhibited the release of malondialdehyde and inflammatory factors including tumor necrosis factor-α and interleukin-6 (all *P* < 0.05). SDSS activates the PI3K-AKT pathway *via* EGFR, promotes endothelial cell migration and angiogenesis, and inhibits oxidative stress and the inflammatory response, highlighting its potential in treating DWs.

## Abstract 2

Sodium Danshensu (SDSS) shows potential in treating diabetic wounds (DWs) owing to its antioxidant, anti-inflammatory, and pro-angiogenesis effects.

## Graphical abstract

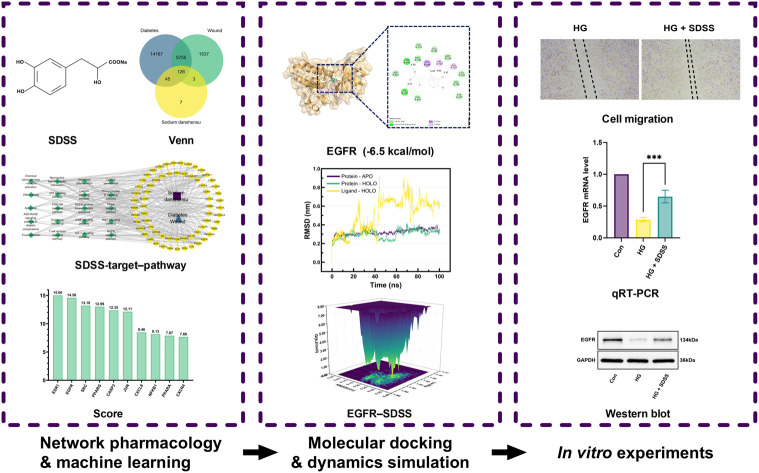

## 1 Introduction

The incidence of diabetic wounds (DWs) is increasing due to an increasing number of patients being diagnosed with diabetes. The International Diabetes Foundation has estimated that 40–60 million people worldwide with diabetes are affected by chronic wounds.^1^ If left untreated, these wounds may lead to soft tissue infection, gangrene, and amputation, or even death,^2^ seriously affecting the quality of life of patients and posing a major global public health challenge. The pathological mechanism of DWs is complex, and disorders in wound healing are closely related mainly to persistent oxidative stress, abnormal inflammatory responses, abnormal cell functions, impaired angiogenesis and cell migration, and disorders of the extracellular matrix.^3–6^ These factors interact to form a “refractory cycle.” Therefore, there is an urgent requirement to identify effective treatment methods.

*Salvia miltiorrhiza*, as a traditional Chinese medicine, has a long history of use in promoting wound healing. The Compendium of Materia Medica included the following indication: For scalds and burns caused by hot oil or fire, use 0.4 kg of *Salvia miltiorrhiza*, finely chopped, mixed with a small amount of water, and decocted in 1.0 kg of mutton fat. Apply the resulting ointment to the injured area.

The main water-soluble component of *S. miltiorrhiza* is Sodium Danshensu (SDSS), which is also known as salvianic acid A sodium or 3,4-dihydroxyphenyllactic acid. It exerts multiple pharmacological effects, such as promoting angiogenesis, inhibiting oxidative stress, and regulating inflammation. In terms of angiogenesis, Jia *et al.* have reported that Danshensu promotes the migration of cerebral microvascular endothelial cells and also lumen formation in rats by activating the PI3K/AKT/mTOR signaling pathway.^7^ Liu *et al.* have confirmed that SDSS can enhance angiogenesis by increasing the levels of hypoxia-inducible factor-1α and vascular endothelial growth factor (VEGF) in cardiac microvascular endothelial cells.^8^ Guan *et al.* have discovered that Danshensu can reverse apoptosis in high glucose-induced endothelial progenitor cells by activating the AKT/eNOS pathway and may also protect from diabetic vasculopathy.^9^ In terms of antioxidant and anti-inflammatory effects, Jia *et al.* have demonstrated that SDSS can alleviate the *tert*-butyl hydroperoxide (*t*-BHP)-induced inhibition of the proliferation of human umbilical vein endothelial cells (HUVECs) in a dose-dependent manner.^10^ Ren *et al.* found that SDSS exerts its anti-inflammatory effects by regulating the macrophage phenotype.^11^ Furthermore, using a pressure ulcer model of rats, Yang *et al.* confirmed that SDSS cream can reduce inflammatory factors and the markers of oxidative stress by regulating the Nrf2/HO-1 and NF-κB pathways, and have demonstrated that it can increase the activities of antioxidant enzymes to promote the healing of pressure ulcers.^12^ However, the above studies have focused on the regulation of endothelial cell dysfunction or local inflammatory wounds (such as pressure ulcers) by SDSS, and there are no studies elucidating the molecular mechanism of SDSS in healing DWs.

Therefore, a combination of network pharmacology, machine learning, molecular docking, molecular dynamics simulation, and *in vitro* experiments was used to systematically identify and verify epidermal growth factor receptor (EGFR) as the core target of SDSS in healing of DWs, revealing that it promotes endothelial cell migration and angiogenesis by activating the PI3K-AKT pathway and inhibiting oxidative stress and inflammatory responses. A multidimensional molecular regulatory network for SDSS in treating DWs was initially constructed, providing a theoretical basis for subsequent in-depth mechanistic research and development of a novel treatment strategy.

## 2 Methods

### 2.1 Materials and reagents

SDSS was purchased from Macklin Biochemical Technology Co., Ltd (Shanghai, China, Lot No. S817894, CAS: 67920-52-9, MDL: MFCD09037393). The molecular formula and molecular weight of SDSS are C_9_H_9_NaO_5_ and 220.15, respectively. Cell counting kit-8 (CCK-8) (Biosharp, Lot No. BL1055A), interleukin-6 (IL-6) assay kit (Bioswamp, Lot No. HM10205), tumor necrosis factor (TNF)-α assay kit (Bioswamp, Lot No. HM10001), catalase (CAT) assay kit (Nanjing Jiancheng, Lot No. A007-1-1), malondialdehyde (MDA) assay kit (Nanjing Jiancheng, Lot No. A003-1-2), lactate dehydrogenase (LDH) assay kit (Nanjing Jiancheng, Lot No. A020-1-2), OSI-774 (Aladdin, Lot No. E408359), LY294002 (MedChemExpress, Lot No. HY-10108) were used. HUVECs were purchased from Zhong Qiao Xin Zhou Biotechnology Co., Ltd (Shanghai, China, Lot No. ZQ1099). This study was conducted in accordance with the principles outlined in the Declaration of Helsinki and was approved by the Medical Ethics Committee of the Chengdu Fifth People's Hospital (No. 2022-020S-01).

### 2.2 Target screening

The target genes of SDSS were identified by searching the SwissTargetPrediction (https://www.swisstargetprediction.ch/) and Super-PRED (https://prediction.charite.de/index.php) databases using the keyword “Sodium Danshensu”. Duplicate entries were removed to obtain the target genes for SDSS. The target genes of DWs were identified by searching the GeneCards (https://www.genecards.org/), OMIM (https://www.omim.org/), and TTD (https://db.idrblab.net/ttd/) databases using the keywords “Diabetes” and “Wound”. Duplicate entries were removed to obtain the target genes for DWs. The intersection of the SDSS and DWs target genes was determined, and a Venn diagram was constructed to visualize the common targets. The overlapping target genes represent the key molecular targets through which SDSS may exert its therapeutic effects in alleviating DWs.

### 2.3 Construction of a protein–protein interaction (PPI) network

The resulting intersection targets were imported into the STRING database (https://string-db.org/), with the species parameter set to “*Homo sapiens*” and other parameters maintained at default values, to construct a PPI network of common target proteins shared between SDSS and DWs. The PPI network diagram was subsequently exported and subjected to further analysis using Cytoscape version 3.9.1.

### 2.4 Gene ontology (GO) function and Kyoto encyclopedia of genes and genomes (KEGG) pathway enrichment analysis

The intersection target data of SDSS and DWs are based on the analysis function of R 4.4.1 clusterProfiler package, including GO function analysis and KEGG path enrichment analysis. The top 10 entries from GO analysis and KEGG analysis were selected for plotting based on the criteria that the difference was statistically significant if *P* < 0.05 was corrected.

### 2.5 Construction of an SDSS-target–pathway network diagram

The SDSS-target–pathway mapping table was established, and Cytoscape 3.9.1 was used to analyze the network topology and construct the SDSS-target–pathway network diagram.

### 2.6 Evaluation and prioritization of the importance of machine learning for biological targets

Data preprocessing, model construction, and validation were performed using Python 3.13.5. First, a scatter plot was constructed based on the network topological features of the targets (degree centrality, betweenness centrality, and closeness centrality), followed by MinMaxScaler normalization. Next, binary classification labels were created based on the 70th percentile, and the data were split into training and test sets in a 7 : 3 ratio. Subsequently, two ensemble models – Random Forest and XGBoost – were employed, with hyperparameter optimization conducted *via* a grid search combined with 5-fold cross-validation. The evaluation further incorporated an additional 10-fold cross-validation to assess stability, and an overfitting analysis was performed by comparing performance differences between the training and test sets. Lastly, to enhance the robustness and stability of the final ranking results, the prediction outputs of RandomForestRegressor and XGBoost regressor were averaged arithmetically to generate an ensemble score, which was then linearly mapped to a 0–15 range to produce the final machine learning score. Complete hyperparameter configurations are provided in SI1, and the machine learning code is available in SI2.

### 2.7 Molecular docking

Molecular docking was used to assess the strength of the bond between the active ingredients and their targets. Based on the ranking of the maximal clique centrality (MCC) algorithm in the PPI network, the top 10 relevant targets were selected for molecular docking with SDSS. The 3D structure of SDSS was downloaded from the PubChem database as an .sdf file. With the key target as the receptor, the .pdb file of the receptor was obtained from the RCSB database. Protein modification–related operations such as dehydration, hydrogenation, and deletion of heteroatoms were performed using PyMOL, and the binding pocket was set using AutoDock. The ability minimization of SDSS was carried out using MMFF94 stance under a pH of 7.4, and rotatable bond angles were set. Subsequently, molecular docking was performed using AutoDock Vina, and the docking scores were recorded.

### 2.8 Molecular dynamics simulation

Molecular dynamics simulations of the target complex system and the apo protein system (APO) were conducted using Gromacs 2023.2. First, the pdb2gmx tool in Gromacs was used to convert the .pdb file into a .gro format coordinate file, and the corresponding topology file (.top) and force field parameter file were generated. The force field selected was AMBER99SB-ILDN, and the small molecule was processed using Acpype for the small molecule GAFF force field, with AM1-BCC charges. Next, the protein–small molecule complex was dissolved using the three-point transferable intermolecular potential solvent, and the minimum distance between the protein atoms and the edge of the water box was at least 1.2 nm. Appropriate amounts of Na^+^ were added to neutralize the charge of the simulation system. Then, the steepest descent algorithm was used for energy minimization to obtain a stable system. The cutoff distance was set to 1.0 nm for short-range electrostatic and van der Waals interactions. The particle mesh Ewald method was used to calculate long-range electrostatic interactions.^13^ The LINCS algorithm was used to constrain all hydrogen bonds that were involved. The Parrinello–Rahman constant pressure regulator was used to maintain a pressure of 1 bar during the simulation, and the V-rescale temperature coupling method was used to adjust the simulation temperature. Subsequently, the protein structure was pre-equilibrated for 100 ps to optimize the initial conformation of the protein in the solvent. In the constant number of particles, volume, and temperature ensemble, it was balanced for 100 ps to warm up the protein and the solvent system to the target temperature. Then, in the constant number of particles, pressure, and temperature (NPT) ensemble, it was balanced for 100 ps to balance the pressure of the solvent and the protein system. After energy minimization and system equilibrium were completed, a 100 ns molecular dynamics simulation was conducted on the protein–small molecule complex using periodic boundary conditions in the NPT ensemble, with a system temperature maintained at 303.15 K and a pressure of 1 bar. The integration time step was 2 fs, and the trajectory (including coordinates, velocities, and energy) was recorded every 10 ps for subsequent analysis. Root mean square deviation (RMSD), root mean square fluctuation (RMSF), solvent accessible surface area (SASA), and radius of gyration (*R*_g_) were calculated using the module in the Gromacs program, and gmx_MMPBSA module combined with free energy and other related calculations were used.

### 2.9 Verification using *in vitro* experiments

#### 2.9.1 Cell culture and treatment

A model of hyperglycemia was established using HUVECs to simulate the microenvironment of diabetes mellitus *in vitro*. Each well was pretreated for 48 h with high-glucose Dulbecco's Modified Eagle Medium (DMEM) having a final concentration of 40 mM glucose. The control group was pretreated with the medium containing the same amount of phosphate-buffered saline (PBS) for 48 h.

#### 2.9.2 CCK-8 assay and determination of LDH activity

The CCK-8 method based on colorimetry was used to determine the effects of different concentrations of SDSS (10, 25, 50, 100, 200 µM) on cell viability, and LDH activity was determined to identify the optimal concentration of SDSS for subsequent experiments. To determine cell viability, cells in the logarithmic growth phase (80% confluent) were digested and centrifuged, adjusted to a concentration of 5 × 10^4^ cells per mL, inoculated on 96-well plates (5000 cells per well), cultured at 37 °C in an atmosphere of 5% CO_2_, and treated for 24 h with different concentrations of SDSS filtered *via* a 0.22 µm membrane. When CCK-8 was detected, the medium was discarded, and the cells were incubated with 1 : 10 CCK-8 reagent for 2 h. The optical density (OD) was measured at 450 nm. To determine LDH activity, cell samples (≥1 × 10^6^) were digested using ultrasound in an ice bath and centrifuged. The supernatant was treated with the components in the kit (buffers, NAD^+^, chromatics, and standards) as follows: Sample/control (50 µL) followed by addition of the buffer (250 µL), incubation at 37 °C for 15 min, addition of NAD^+^ (50 µL) and the coloring agent (250 µL), incubation for a further 15 min, and addition of 0.4 M NaOH (2.5 mL) for color development for 5 min. The OD was measured at 440 nm. Cell viability was calculated based on the OD value, and LDH activity was quantitatively analyzed using the standard curve.

#### 2.9.3 Assessment of angiogenesis

##### 2.9.3.1 Determination of cell migration

Cell migration was assessed using a standardized scratch assay. First, an even horizontal line was drawn on the back of a 6-well plate to ensure accurate positioning. Subsequently, cells in the logarithmic growth phase were digested into a single-cell suspension. Next, 5–10 × 10^5^ cells were transferred to each well. Individual cell plates were cultured to complete fusion, and the culture medium was replaced with the drug-containing medium for a specified time. A 200 µL vertical scratch was made with a pipette tip and then rinsed with PBS to remove the shed cells. Images were taken at a fixed position at 0 h, 12 h, 24 h, and 48 h, with 3 fields of view recorded for each time point. ImageJ software was used to analyze the intercellular distance. Cell mobility (wound healing rate) = (mean initial intercellular distance − mean intercellular distance at time *T*)/mean initial intercellular distance.

##### 2.9.3.2 Tube-formation assay

Matrigel-based matrix gel tube-formation assay was used to evaluate *in vitro* angiogenesis. Cells in the logarithmic growth phase (passages 2–6) were trypsinized, counted, and seeded at optimized densities into culture plates and incubated overnight. Subsequently, drug-containing medium was added for specific durations to establish the model. Prior to the experiment, Matrigel was thawed overnight at 4 °C in an icebox, whereas serum-free medium, pipette tips, and plates were prechilled. All experiments were performed on ice to prevent denaturation. Matrigel was evenly spread onto the plates (avoiding bubbles) and polymerized for 1 h at 37 °C in an incubator flushed with 5% CO_2_. After treatment, the cells were digested, centrifuged, resuspended in serum-free medium, and plated onto the solidified gel. After incubation at 37 °C in the presence of 5% CO_2_, images were captured at 0, 5, and 10 h using microscopy. Vascular network formation was visible between 3 and 12 h. Lastly, images at the 5-h timepoint were analyzed using ImageJ software to quantify the total tube length and branch points and to assess the tubular structure–formation capacity.

#### 2.9.4 Determination of anti-inflammatory and antioxidant effects

IL-6 and TNF-α levels in the cell supernatants were quantitatively analyzed using enzyme-linked immunosorbent assay (ELISA). Standard solutions were serially diluted (6 concentration gradients). Samples and biotin-labeled antibodies were added in a 40 : 10 µL ratio and incubated at 37 °C for 30 min. After 5 washes, 50 µL of chromogenic solutions A/B were each added for color development for 10 min while protecting from light. OD values were measured at 450 nm, and the concentrations of IL-6 and TNF-α were calculated using the standard curve. The levels of the antioxidant stress factors CAT and MDA in cells were determined using the corresponding assay kits. MDA determination required 40 min of color development at 95 °C and measurement of the OD at 532 nm using colorimetry. MDA levels were calculated based on the standard curve of tetraethoxypropane as follows: MDA content = (sample OD − control OD)/(standard OD − blank OD) × 10 nmol mL^−1^/protein concentration. The change in the absorbance in the reaction system at 405 nm was monitored within 1 min (CAT activity = Δ*A* × 235.65/0.1 mL/60 s/protein concentration). All samples were centrifuged at 2000–3000 rpm for 20 min and stored at −80 °C to avoid freezing and thawing.

#### 2.9.5 Quantitative real-time polymerase chain reaction (qRT-PCR)

Gene expression was determined using qRT-PCR. Primer design specifications were as follows: mRNA/lncRNA (18–25 bp, GC content 45–55%), miRNA (stem-loop), and circRNA (backsplice). RNA extraction involved purification with TRIzol, and 500 ng of total RNA was used for reverse transcription as a template. qPCR and detection using SYBR Green was performed under the following conditions: pre-denaturation at 95 °C, followed by 40 cycles of 95 °C/60 °C. Data were analyzed using the ΔΔCt method (2^−ΔΔCt^) normalized to the housekeeping gene GAPDH. Primer sequences are presented in Table 1.

**Table 1:** Sequences of primers for qRT-PCR

Gene		Sequences (5′ → 3′)	Length (bp)	*T*_m_ (°C)
GAPDH	Forward	CAGGAGGCATTGCTGATGAT	138	60
	Reverse	GAAGGCTGGGGCTCATTT		60
EGFR	Forward	ATGGCCAGCGTGGACAACCC	152	66
	Reverse	TTGAGCAGGTACTGGGAGCC		62

#### 2.9.6 Western blotting

The expression of target proteins was determined using western blotting. Protein extraction was performed using RIPA lysis buffer (containing protease/phosphatase inhibitors). Adherent cells were lysed after washing with TBS, whereas cell suspensions and tissue samples were homogenized. The supernatant was obtained by centrifugation at 12 000 rpm for 5 min at 4 °C. Protein samples were mixed with 2× loading buffer and denatured at 100 °C for 5 min. A 10% separating gel and 5% stacking gel (80 V for the stacking gel → 130 V for the separating gel) was used for electrophoresis. The protein bands were electro-transferred to a polyvinylidene fluoride membrane at 250 mA for 1 h on ice. The membrane was blocked with 5% skim milk for 1 h, followed by overnight incubation with the primary antibody at 4 °C and incubation for 1 h with the secondary antibody at room temperature, with 5 washes with TBST each time. Enhanced chemiluminescence detection was used, and the gray values of the protein bands were analyzed using ImageJ.

### 2.10 Statistical analysis

All quantitative experiments were independently performed at least 3 times, and experimental data are expressed as mean ± standard deviation. Statistical analysis was performed using GraphPad Prism 10.1.2, and differences between groups were compared using one-way analysis of variance and Tukey's *post hoc* test for multiple comparisons. Results are expressed as mean difference (MD) and 95% confidence intervals (CIs). Differences were considered significant at *P* < 0.05.

## 3 Results

### 3.1 Target results

**Fig. 1:**
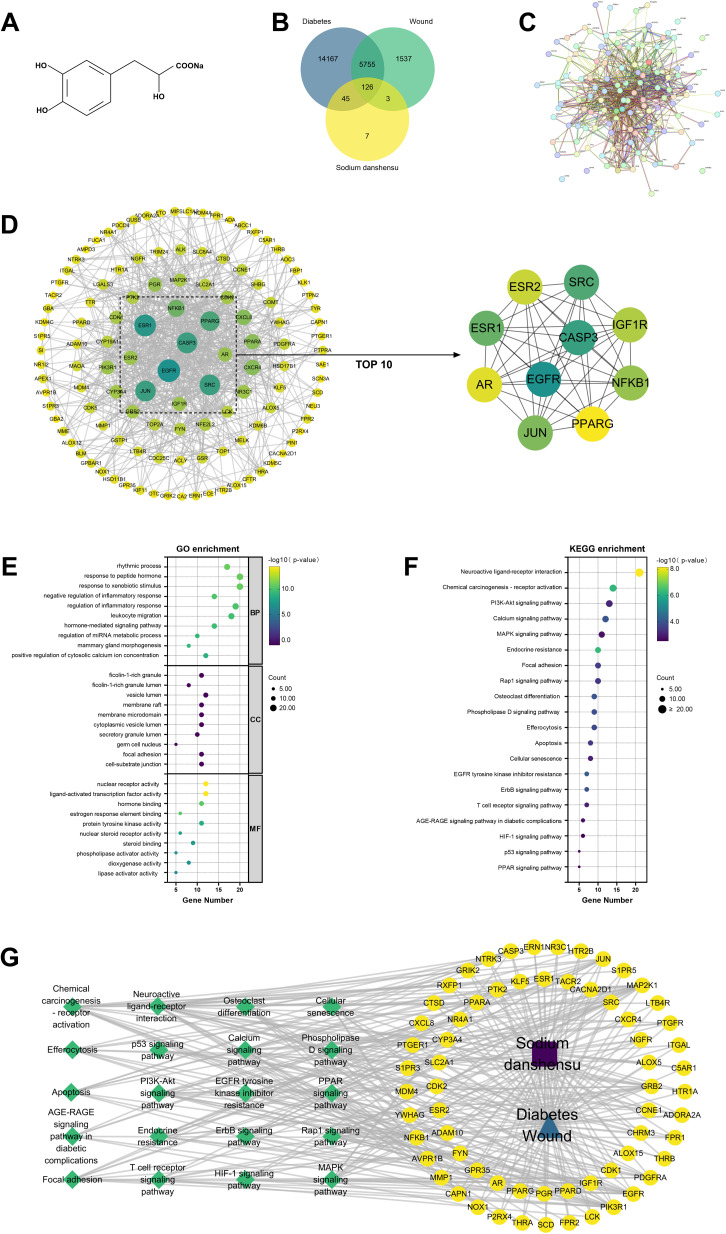
Network pharmacology analysis of SDSS treatment for DWs. (A) The structural formula of SDSS. (B) Venn diagram of SDSS-regulated DWs targets. (C) PPI network diagram. (D) Optimized PPI network diagram and the MCC algorithm was used to obtain the top ten targets. The size and color of the nodes are proportional to the number of edges. Larger nodes and darker colors indicate stronger interactions, suggesting that these interactions play a decisive role in the PPI network. (E) GO enrichment analysis of SDSS and DWs targets (Top 10). (F) KEGG enrichment analysis of SDSS and DWs targets (Top 20). (G) Network of the main pathways and targets of SDSS treatment of DWs.

The structural formula of SDSS is shown in Fig. 1A. Using the GeneCards, OMIM, and TTD databases, the keywords “Diabetes” and “Wound” were searched, and 20 093 and 7421 disease targets were obtained, respectively. The target of SDSS was searched using the SwissTargetPrediction and Super-PRED databases, and 181 drug targets were obtained after sorting. The intersection of drug targets and disease targets yielded 126 targets, as shown in the Wenn diagram (Fig. 1B). The complete target data can be found in SI3.

### 3.2 PPI network analysis

The above repeated targets were input into the STRING database to obtain the PPI network diagram (Fig. 1C), and the MCC algorithm was used to obtain the top 10 targets, namely EGFR, CASP3, SRC, ESR1, JUN, NFKB1, IGF1R, ESR2, AR, and PPARG (Fig. 1D).These nodes may play a key role in the entire network and are the key targets of SDSS intervention in alleviating DWs.

### 3.3 GO/KEGG enrichment analyses

Results from GO enrichment analysis revealed a total of 880 biological processes (BPs), 35 cellular components (CCs), and 118 molecular functions (MFs). The top 10 items of each module are listed according to *P*-value (Fig. 1E). Ninety-four pathways were obtained after KEGG pathway enrichment analysis (*P* < 0.05; Fig. 1F). The identified BPs included rhythmic process, response to peptide hormone, and response to xenobiotic stimulus; CCs mainly involved ficolin-1 – rich granule, ficolin-1 – rich granule lumen, and vesicle lumen. MFs mainly included nuclear receptor activity, ligand-activated transcription factor activity, and hormone binding, suggesting that SDSS may interfere with DWs *via* these processes. KEGG pathway enrichment analysis revealed that the targets of SDSS were closely related to the interaction of the neuroactive ligand–receptor interaction, chemical carcinogenesis–receptor activation, and PI3K-AKT signaling pathway, suggesting that the active components of SDSS may play a crucial role in alleviating DWs by controlling several biological pathways.

### 3.4 SDSS-target–pathway diagram

The SDSS-target–pathway diagram was constructed using Cytoscape 3.9.1. In this network, the targets MAP2K1, PIK3R1, EGFR, GRB2, JUN, NFKB1, PDGFRA, and IGF1R exhibit more connected edges than the other targets and are more correlated, suggesting an important role in this network and highlighting them as key targets of SDSS in interfering with DWs. Moreover, the interactions between pathway neuroactive ligand–receptor interaction, chemical carcinogenesis–receptor activation, PI3K-AKT signaling pathway, calcium signaling pathway, and MAPK signaling pathway also had a considerable involvement in the network, which may play an important role in the effect of SDSS in alleviating DWs (Fig. 1G).

### 3.5 Target results from machine-learning optimization

**Fig. 2:**
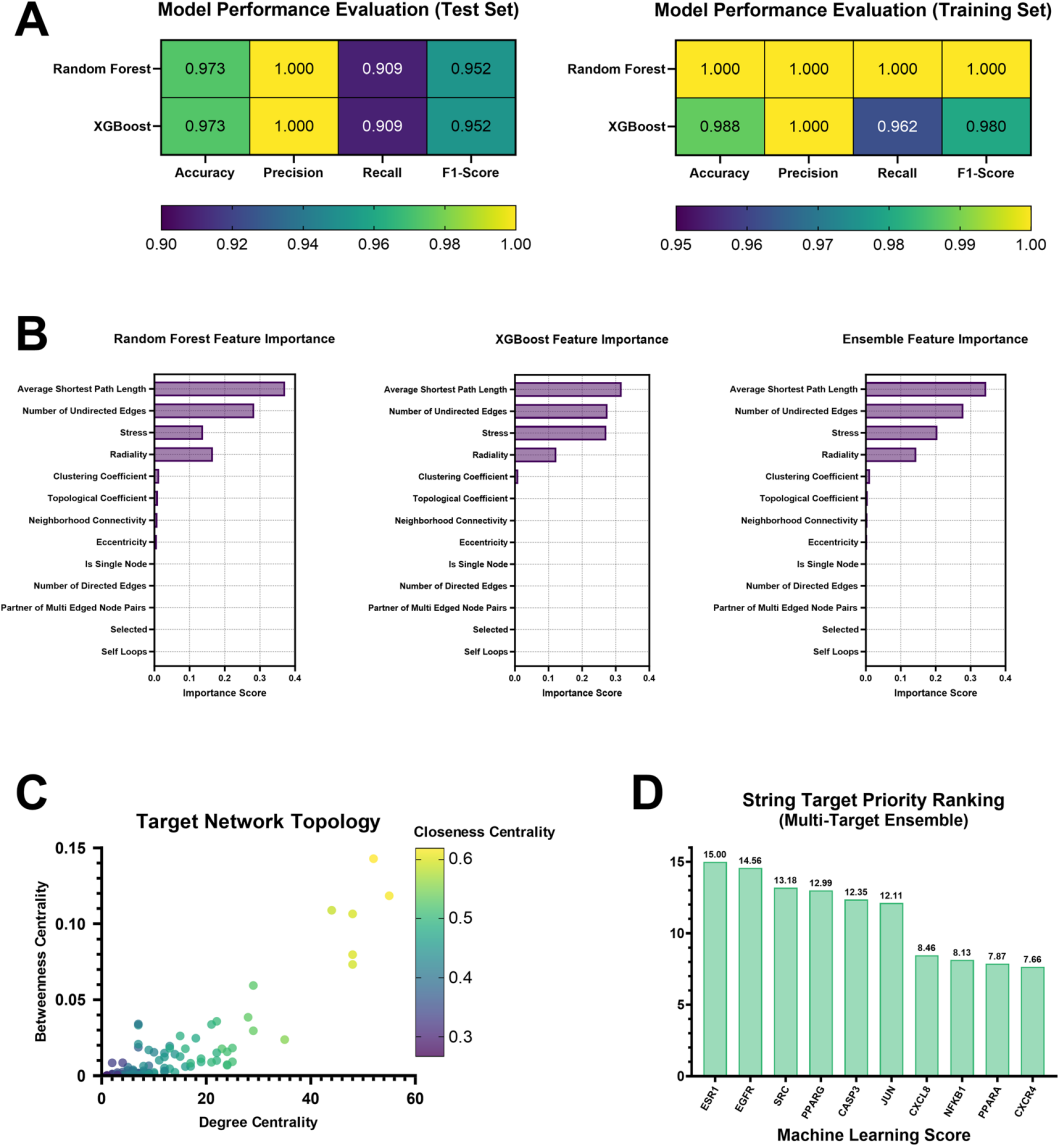
Machine learning optimization target results. (A) Heatmaps for model performance metrics. (B) Feature importance score. (C) Network topological feature scatter plot. (D) Top 10 machine learning score targets.

After hyperparameter optimization, both RandomForest and XGBoost classifiers achieved 97.3% accuracy and 95.2% F1-score on the test set; the mean F1-scores from 10-fold cross-validation were 0.960 and 0.980, respectively, with low standard deviations, indicating model robustness. Overfitting analysis revealed that the differences between training and test accuracy were both <0.05 (RandomForest: 0.027, XGBoost: 0.015), whereas F1-score differences were 0.048 and 0.028, respectively, indicating a low risk of overfitting. Heatmaps were generated for model performance metrics (Fig. 2A). The models achieved excellent performance with *R*^2^ > 0.96 in the regression task. The top 5 features by importance were average shortest path length, number of undirected edges, stress, radiality, and clustering coefficient (Fig. 2B). A scatter plot based on network topological features was generated (Fig. 2C). In the final integrated target prioritization ranking, ESR1, EGFR, and SRC were identified as the highest-priority targets (Fig. 2D). Training-testing performance comparison and 10-fold cross-validation results are presented in SI1.

### 3.6 Molecular docking

**Fig. 3:**
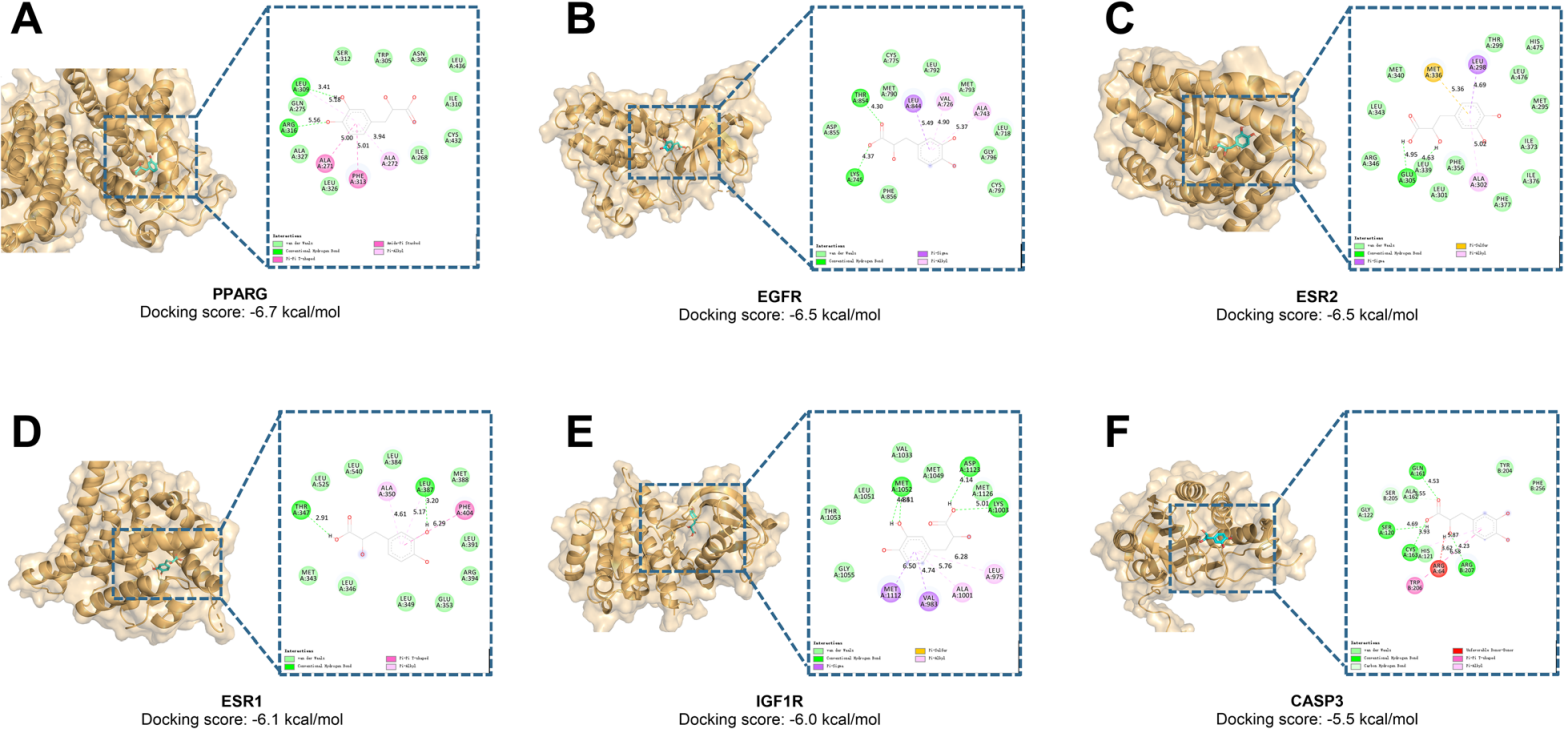
Molecular docking results between core targets and components. (A–F) Molecular docking presentations of the top six pairs with the best binding energy.

Molecular docking of the top 10 key targets selected above and SDSS was performed using AutoDock. Chemical structures of the compounds were downloaded from the PubChem platform, and protein files were downloaded from the PDB database. SDSS and the 10 targets were found to bind well (Table 2). The binding energy of the compound and the target was less than −4.3 kcal mol^−1^, and the lowest energy was −6.7 kcal mol^−1^, indicating these proteins to be potential binding targets of SDSS. The first 6 results with the lowest binding energies were analyzed (Fig. 3A–F), and the volume information of the grid boxes is presented in SI1.

**Table 2:** Top 10 target blind energy results

Component	Target	Binding energy (kcal mol^−1^)
Sodium Danshensu	PPARG	−6.7
	EGFR	−6.5
	ESR2	−6.5
	ESR1	−6.1
	IGF1R	−6.0
	CASP3	−5.5
	JUN	−5.3
	SRC	−4.6
	AR	−4.3
	NFKB1	−4.3

### 3.7 Molecular dynamics simulation

**Fig. 4:**
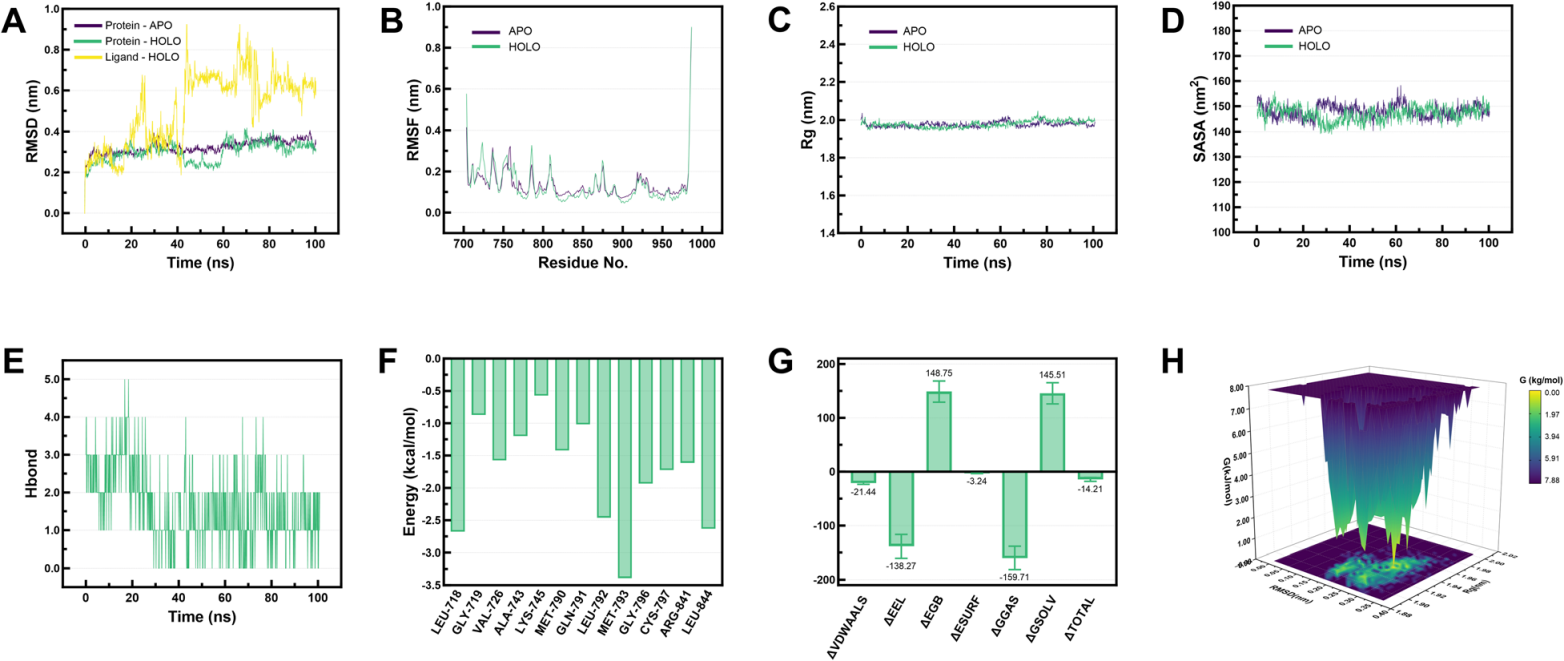
Molecular dynamics simulation of 100 ns. (A) RMSD. (B) RMSF. (C) *R*_g_. (D) SASA. (E) Hydrogen bond. (F) Binding free energy. (G) Residue decomposition of binding free energy. (H) Free energy landscape. HOLO: EGFR–SDSS system; APO: apo protein system.

**Fig. 5:**
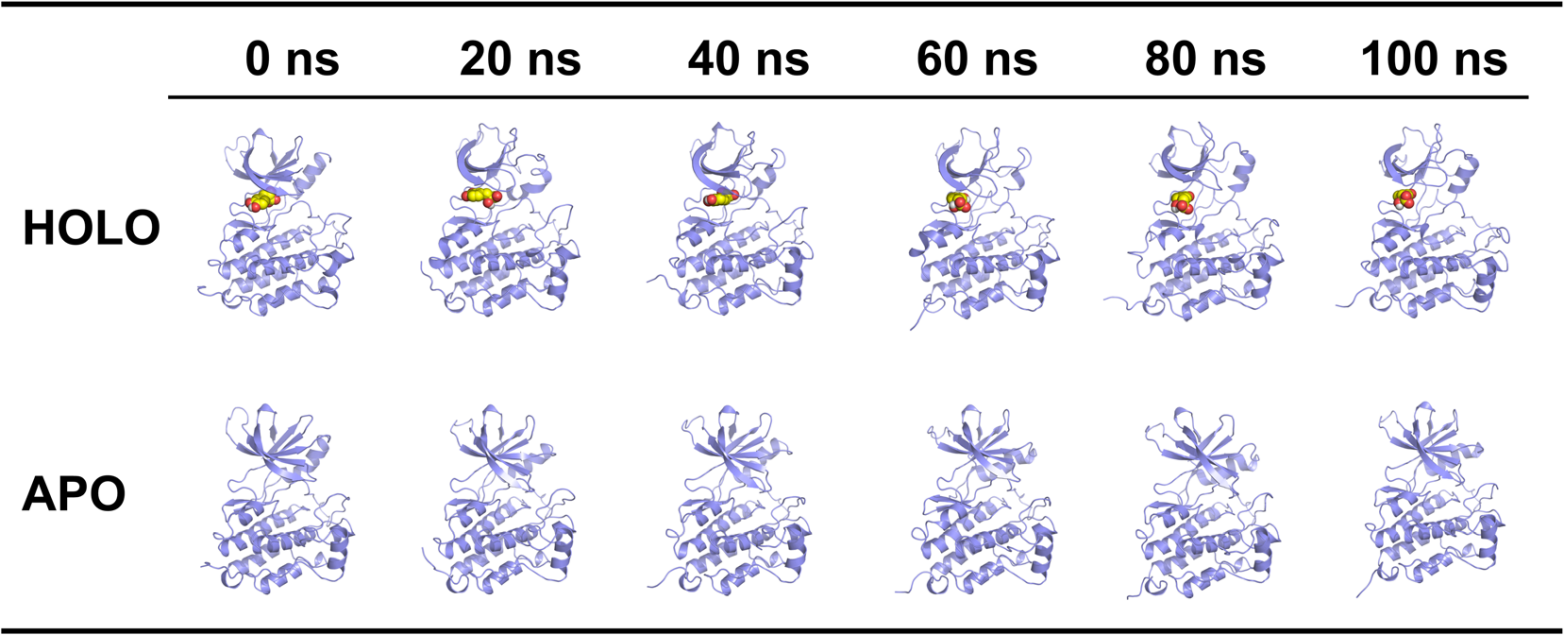
The trajectory snapshot of 0–100 ns of the target complex system and the app protein system. HOLO: EGFR–SDSS system; APO: apo protein system.

Based on the results of the evaluation of the multidimensional algorithm, EGFR demonstrated potential as a core target of SDSS in regulating healing of DWs. Based on these findings, a 100 ns molecular dynamics simulation of the EGFR–SDSS system (HOLO) was performed, with the unbound SDSS APO serving as a control. The RMSD of HOLO stabilized at approximately 0.3 nm after 60 ns, whereas APO showed a continuous upward trend. The RMSD of the ligand (SDSS) tended to 0.6 nm after 70 ns, indicating that its binding conformation gradually converged (Fig. 4A). Except for a few sites, the amino acid fluctuations in HOLO were all <0.5 nm, and the fluctuation amplitudes of multiple domains were lower than those of APO (Fig. 4B). SASA and *R*_g_ showed minor differences between the two systems (Fig. 4C and D). EGFR and SDSS could form hydrogen bonds; the number of hydrogen bonds was mostly between 0 and 3, and the hydrogen bond interaction was persistent (Fig. 4E). MET-793/LEU-718/LEU-844 was found to play a key role in the interaction between EGFR and SDSS, and the binding free energy for EGFR and SDSS was −14.21 ± 3.61 kcal mol^−1^ (Fig. 4F and G). HOLO had a dominant complex conformation with an RMSD of 0.15–0.30 nm and an *R*_g_ of 1.89–1.96 nm, and the structure was more stable (Fig. 4H). Fig. 5 shows the trajectory snapshot of 0–100 ns of HOLO and APO.

### 3.8 *In vitro* experiments

#### 3.8.1 Screening to determine the optimal concentration and experimental groups

**Fig. 6:**
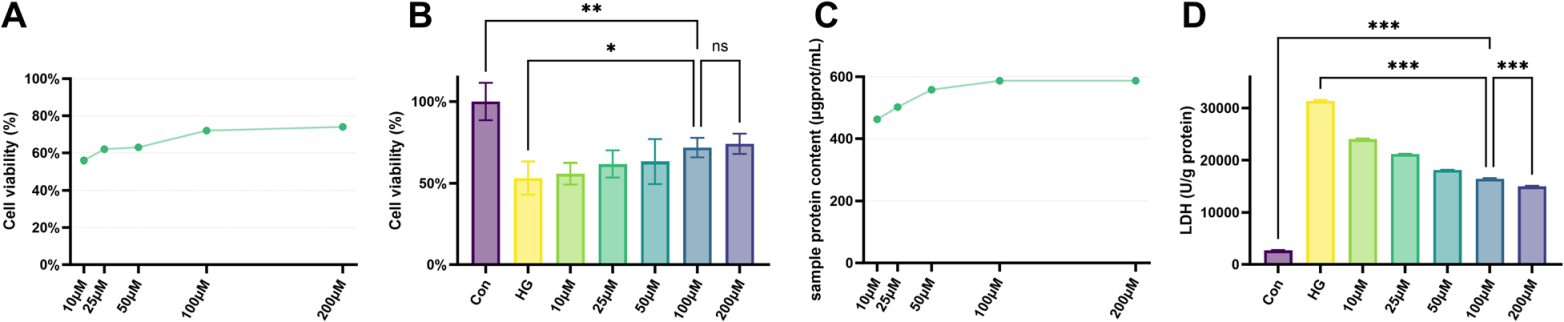
Optimal concentration screening of SDSS. (A and B) Effect of different concentrations of SDSS solution on HUVEC viability. (C) Protein concentration in samples measured by bicinchoninic acid assay after treatment with various SDSS concentrations. (D) LDH activity in HUVECs exposed to different SDSS concentrations. Con: control group; HG: high-glucose group (*P < 0.05, **P < 0.01, ***P < 0.001.)

Quantitative analysis using the CCK-8 and LDH assays revealed the concentration-dependent effect of SDSS on the cytotoxicity and proliferation of HUVECs (10–200 µM). Furthermore, no significant difference in cell viability was noted at concentrations of 100 µM and 200 µM (MD and 95% CIs = −2.34 [−23.77, 19.09], *P* = 0.999). Accordingly, 100 µM was selected as the optimal concentration for subsequent experiments (Fig. 6A–D). The experimental groups comprised the control group, which was cultured in standard medium with PBS; the high-glucose (HG) group, which received DMEM containing 40 mM glucose; the HG + SDSS group, which was exposed to high-glucose medium supplemented with 100 µM SDSS; the HG + SDSS + LY group, which was treated with high-glucose medium containing both 100 µM SDSS and LY294002; and the HG + SDSS + OSI group, which was treated with high-glucose medium containing both 100 µM SDSS and OSI-774.

#### 3.8.2 Regulation of SDSS in angiogenesis function

**Fig. 7:**
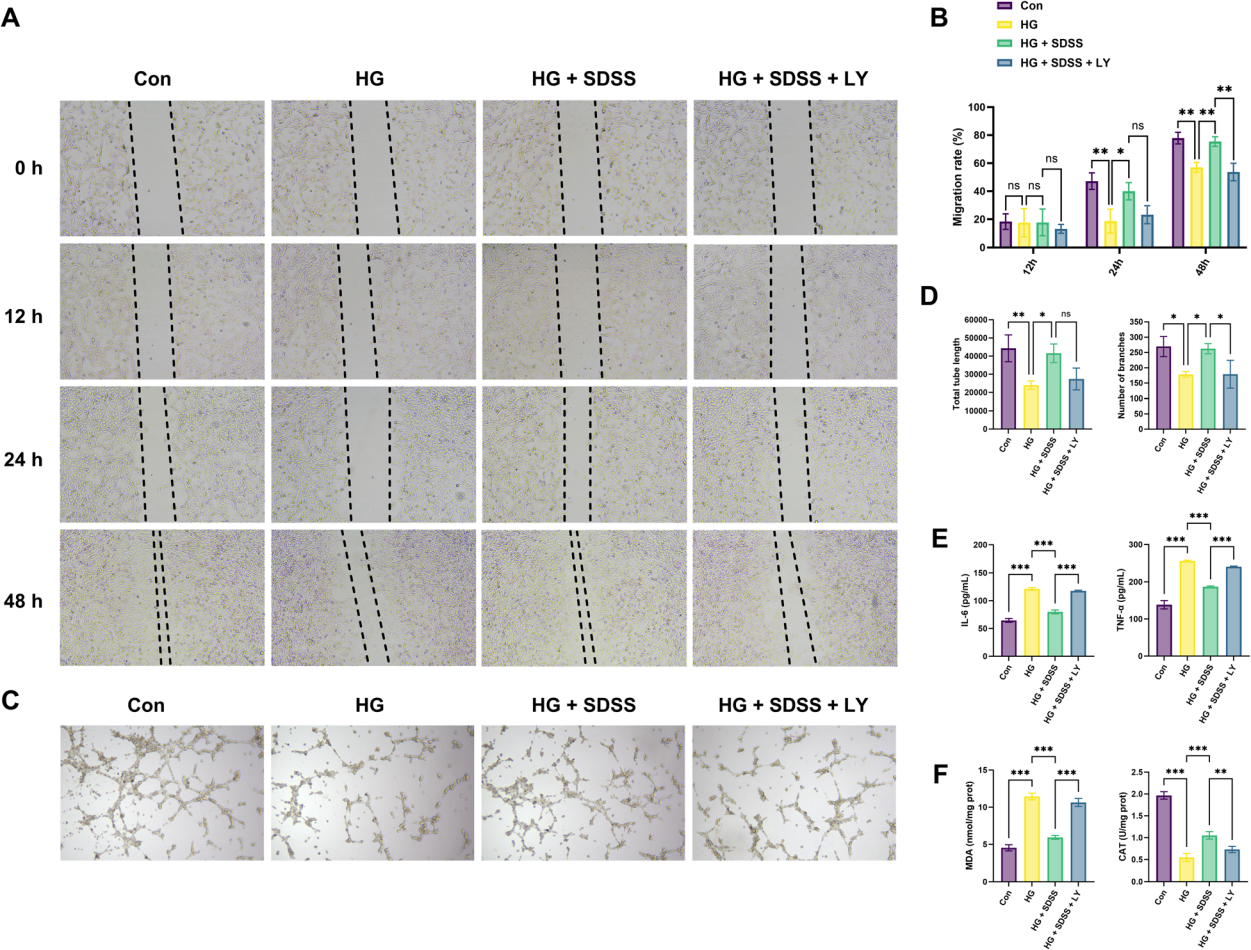
SDSS promotes the migration and angiogenesis of HUVEC cells, and inhibits oxidative stress and inflammation. (A and B) Scratch wound healing assay in different groups and the change of results. (C and D) Tube formation assay results evaluating angiogenic capacity in different groups. (E) Measurement of IL-6 and TNF-α levels in different groups. (F) Measurement of MDA and CAT activity in different groups. Con: control group; HG: high-glucose group; HG + SDSS: high-glucose + 100 µM SDSS group; HG + SDSS + LY: high-glucose + 100 µM SDSS + LY294002 group (*P < 0.05, **P < 0.01, ***P < 0.001.)

Findings from the scratch assay revealed the wound-healing rate in the control group to be higher than that in the HG group for 24 and 48 h, whereas the healing rate in the HG + SDSS group demonstrated an intermediate value between the two (Fig. 7A). Quantitative analysis at 48 h revealed that exposure to high glucose levels reduced the mobility of cells in the HG group compared with those in the control group (−20.86 [−32.57, −9.16], *P* < 0.01), whereas SDSS intervention significantly improved the mobility of cells in the HG + SDSS group (18.40 [6.69, 30.10], *P* < 0.05, Fig. 7B). Findings from the Matrigel tube-formation experiment indicated that the total lumen length and the number of branch nodes in the control group were higher than those in the HG group (20 240.00 [5814.00, 34 666.00]; 91.00 [13.29, 168.70], all *P* < 0.05). After SDSS intervention, the lumen length and the number of branches increased in the HG + SDSS group *versus* those in the HG group (17 527.00 [3101.00, 31 953.00]; 84.00 [6.29, 161.70], all *P* < 0.05; Fig. 7C and D).

#### 3.8.3 Effects of SDSS on inflammation and oxidative stress

Results from ELISA revealed that the secretion of IL-6 and TNF-α in the HG group was significantly higher than that in the control group (56.62 [51.62, 61.62]; 118.40 [107.80, 129.00], all *P* < 0.001). After SDSS intervention, the levels of these inflammatory cytokines were lower in the HG + SDSS group than in the HG group (−41.47 [−46.47, −36.47]; −69.88 [−80.49, −59.28], all *P* < 0.001, Fig. 7E). Biochemical analyses revealed a significant increase in MDA levels and a decrease in CAT activity (6.91 [5.81, 8.01]; −1.42 [−1.64, −1.20], all *P* < 0.001) in the HG group *versus* those in the control group, whereas SDSS intervention significantly decreased MDA levels and increased CAT activity in the HG + SDSS group compared with those noted in the HG group (−5.51 [−6.61, −4.40]; 0.51 [0.29, 0.72], all *P* < 0.001, Fig. 7F).

#### 3.8.4 EGFR-mediated PI3K-AKT pathway regulates migration, angiogenesis, inflammation, and oxidative stress in HUVECs

**Fig. 8:**
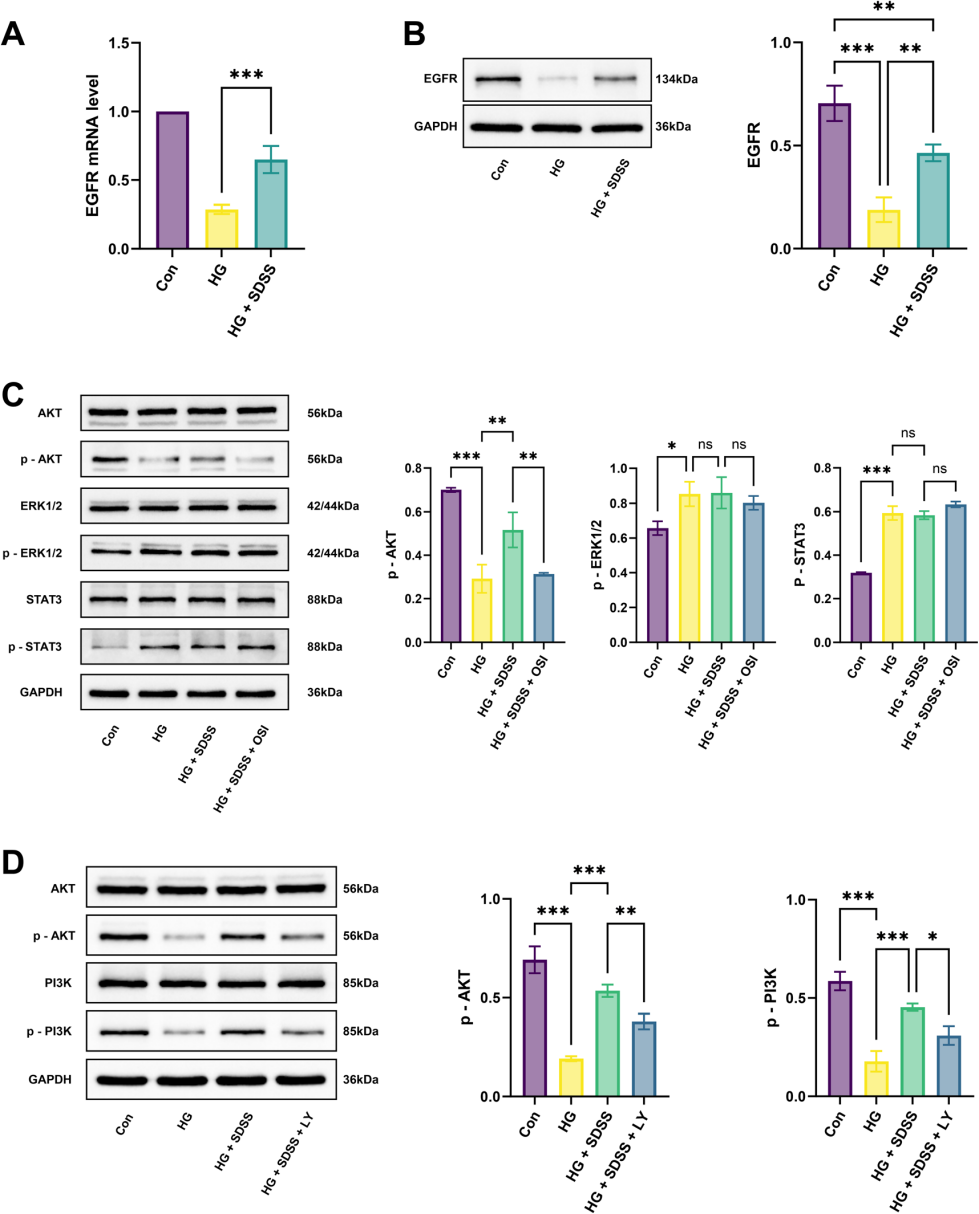
Potential role and mechanism of SDSS treatment for DWs. (A) The gene expression of EGFR in different groups. (B) The protein expression of EGFR in different groups. (C) The protein expression of AKT, p-AKT, ERK1/2, p-ERK1/2, STAT3, and p-STAT3 in different groups. (D) The protein expression of AKT, p-AKT, PI3K, and p-PI3K in different groups. Con: control group; HG: high-glucose group; HG + SDSS: high-glucose + 100 µM SDSS group; HG + SDSS + OSI: high-glucose + 100 µM SDSS + OSI-774 group; HG + SDSS + LY: high-glucose + 100 µM SDSS + LY294002 group (*P < 0.05, **P < 0.01, ***P < 0.001.)

EGFR was used as the core target to construct an *in vitro* validation system. Findings from qRT-PCR and western blotting indicated that the mRNA expression of EGFR in the HG + SDSS group was significantly higher than that in the HG group (0.36 [0.21, 0.51], *P* < 0.001; Fig. 8A), with protein expression exhibiting a similar trend (0.28 [0.11, 0.44], *P* < 0.01; Fig. 8B). To explore the key downstream pathways of EGFR signal transduction, potential pathways such as PI3K-AKT and MAPK were screened based on KEGG enrichment analysis. Preliminary verification was conducted using the EGFR inhibitor OSI-774 (erlotinib). The protein expression of p-AKT in the HG + SDSS group was higher than that in the HG group (0.14 [0.03, 0.25], *P* < 0.05), whereas that of p-ERK1/2 and p-STAT3 was not significantly different between the HG and the HG + SDSS groups (0.01 [−0.09, 0.10]; 0.01 [−0.04, 0.06], all *P* > 0.05). After treatment with the EGFR inhibitor, the protein expression of p-AKT in the HG + SDSS + OSI group was lower than that in the HG + SDSS group (−0.20 [−0.31, −0.09], *P* < 0.01; Fig. 8C), indicating that the PI3K-AKT pathway may be the key downstream signaling pathway of EGFR. Pathway intervention experiments were conducted to further elucidate the role of the PI3K-AKT pathway in EGFR signal transduction. After treatment with the PI3K inhibitor LY294002, the protein expression of p-PI3K and p-AKT in the HG + SDSS + LY group decreased significantly compared with that recorded for the HG + SDSS group (−0.14 [−0.25, −0.03]; −0.15 [−0.27, −0.04], all *P* < 0.05; Fig. 8D). Moreover, except for the total length of the lumen (−14165.00 [−28591.00, 261.00], *P* = 0.05), other parameters such as the cell-migration ability (48 h), lumen-formation ability (number of branch nodes), and inflammatory/oxidative stress indices in the HG + SDSS + LY group showed a significant reduction (all *P* < 0.05; Fig. 7A–F).

## 4 Discussion

The multipathway mechanisms by which SDSS promotes the healing of DWs were systematically elucidated in this study using a combination of network pharmacology, machine learning, molecular dynamics simulations, and *in vitro* experiments. This multiomics strategy not only addresses the limitations of traditional methods but also highlights the molecular basis of the therapeutic effects of SDSS of targeting the EGFR and regulating critical signaling pathways including the PI3K-AKT pathway. Delayed healing of DWs is a common outcome of endothelial dysfunction, impaired angiogenesis, inflammatory responses, and oxidative stress, all of which can be therapeutically modulated by targeting these pathways.^14^ The combination of network pharmacology predictions and machine learning further improves the accuracy of target screening, providing a theoretical basis for the clinical applications of SDSS.

Multidimensional algorithm evaluations revealed EGFR to be the core target in SDSS-mediated healing and regulation of DWs, ranking it first in the MCC algorithm and second in both molecular docking binding energy and machine-learning scores. EGFR is a member of the ErbB receptor tyrosine kinase family, comprising 3 critical domains: an extracellular ligand-binding region, a transmembrane domain, and an intracellular kinase domain. It regulates cell proliferation, differentiation, and apoptosis by activating downstream signaling pathways such as the MAPK and PI3K/AKT pathways.^15^ EGFR promotes wound healing through the following mechanisms: (1) regulating cell growth and proliferation: upon ligand binding, EGFR activates its intracellular tyrosine kinase domain, initiating downstream signaling pathways that promote cell cycle progression and DNA synthesis; (2) promoting cell differentiation and migration: EGFR induces cell differentiation during embryonic development and tissue repair, enhancing the migratory capacity of cells; (3) maintaining cell survival: by activating anti-apoptotic signaling pathways, EGFR reduces cell apoptosis; (4) participating in tissue repair: under normal physiological conditions, EGFR contributes to processes such as wound healing and mucosal repair.^16^

In the 100 ns molecular dynamics simulation, the RMSD of HOLO stabilized after 60 ns, whereas the unbound SDSS APO continued to show an upward trend. This phenomenon indicated that the ligand binding of SDSS enhanced the overall structural stability of the EGFR protein. Further analysis at the residue level revealed that the fluctuation amplitudes of multiple domains in HOLO were lower than those in APO, suggesting that the conformational stability of the amino acid residues of the protein improved after complex formation. This finding further confirmed that the conformational binding of SDSS with EGFR was not only stable but also had a higher stability than the EGFR state without ligand binding. In terms of system compactness, the *R*_g_ of HOLO was approximately 1.9 nm and the SASA was 150 nm^2^, indicating the structure of this complex to be compact and the solvent-exposed surface to be stable. Interaction analysis revealed the formation of a continuous hydrogen bond network (0–3) between SDSS and EGFR and indicated that these hydrogen bonds played a key role in contributing to the stable binding of the complex. The free-energy calculation results indicated the binding free energy of SDSS with EGFR to be −14.21 ± 3.61 kcal mol^−1^, indicating the binding process to be thermodynamically feasible. Further analysis revealed that the key residues MET-793, LEU-718, and LEU-844 played a guiding role during the binding process, possibly stabilizing the conformation of the complex *via* hydrophobic or other interactions.^17,18^ Free energy profile analysis indicated that conformations with a relative deviation value of 0.15–0.30 nm and *R*_g_ of 1.89–1.96 nm dominated during the simulation and that these conformations exhibited the lowest binding free energy and the highest stability. This result was consistent with the RMSD and residue fluctuation analysis, further supporting the stability advantage of the SDSS–EGFR complex conformation. Overall, SDSS could stably bind to EGFR, and the participation of key residues and the joint action of the hydrogen bond network maintained the conformational stability of the complex. Moreover, the binding of SDSS may enhance the reliability of target selection by optimizing its binding pose with EGFR, providing a structural basis for subsequent drug design.

The pathological mechanisms underlying DWs recalcitrance primarily involve chronic inflammation and an imbalance in oxidative stress. Under high-glucose conditions, macrophage dysfunction manifests as impaired phagocytic capacity, reduced polarization from the pro-inflammatory M1 to the reparative M2 phenotype, and a significantly increased secretion of pro-inflammatory cytokines including IL-6 and TNF-α.^19^ Our findings revealed that SDSS could ameliorate this inflammatory state by reducing the high-glucose-induced increase in IL-6 and TNF-α levels, a result that was consistent with the findings by Zhang *et al.* that SDSS can suppress macrophage inflammation *via* the miR-200a-3p/MEKK3/NF-κB signaling pathway.^20^ Furthermore, DWs are characterized by severe oxidative stress, the excessive accumulation of reactive oxygen species such as superoxide anion radicals and hydroxyl radicals, and the inhibition of antioxidant enzymes including CAT.^21^ Our findings confirm the dual antioxidant effects of SDSS, *i.e.*, reducing MDA levels and enhancing CAT activity, which align with the results reported by Jia *et al.* that SDSS can regulate antioxidant enzyme expression *via* the PI3K/AKT/GSK-3β pathway and inhibit the *t*-BHP-induced increase in MDA in HUVEC.^10^ Another critical pathological mechanism involves impaired endothelial cell migration and angiogenesis. Our findings suggested that SDSS increased the migration of HUVECs in high-glucose conditions in scratch assays, whereas, in tube formation assays, the total lumen length and branch node counts also increased, indicating the role of SDSS in promoting cell migration and tubular structure formation to improve angiogenesis. Overall, SDSS exhibited anti-inflammatory, antioxidant, pro-migratory, and pro-angiogenic effects in HUVECs exposed to high-glucose conditions, highlighting its potential in treating DWs.

KEGG enrichment analysis revealed the key signaling pathways through which SDSS acts on DWs. These pathways included neuroactive ligand–receptor interactions, chemical carcinogenesis–receptor activation, PI3K-AKT signaling pathway, calcium signaling pathway, and MAPK signaling pathway. Combined with the previously locked EGFR target, as a member of the protein tyrosine kinase family, EGFR may play a pivotal role in the upstream regulatory network of PI3K-AKT, MAPK, and JAK-STAT.^22–24^ Findings from qRT-PCR and western blotting indicated that SDSS could upregulate EGFR expression. When exploring the key downstream pathways mediated by EGFR, SDSS was found to upregulate the protein expression of p-AKT in HUVECs under high glucose conditions, and the protein expression of p-AKT decreased after adding the EGFR inhibitor OSI-774. However, the protein expression of p-ERK1/2 and p-STAT3 in HUVECs under high glucose conditions did not change significantly after SDSS intervention, indicating the PI3K-AKT pathway as the key downstream signaling pathway of EGFR. When the PI3K inhibitor LY294002 was used, the protein expression of p-PI3K/p-AKT decreased, and the migration ability, lumen-formation ability, and inflammatory/oxidative stress indicators of HUVECs also demonstrated a decrease. These findings further confirmed the core role of this pathway. The PI3K-AKT pathway is crucial for the healing of DWs; its activation accelerates wound healing by promoting angiogenesis, mitigating oxidative stress, and suppressing inflammation.^25,26^ This mechanism parallels the effects of traditional Chinese medicine formulations such as Shengji-Huayu that promote endothelial function and wound repair by modulating the PI3K-AKT pathway.^27,28^ As an RTK family member, EGFR is an upstream signaling molecule of the PI3K-AKT pathway. Its activation recruits and activates PI3K, converting phosphatidylinositol 4,5-bisphosphate to phosphatidylinositol 3,4,5-trisphosphate, thereby activating AKT and influencing the survival, proliferation, migration, and angiogenesis of cells.^29–31^

Our study has some limitations. First, high-glucose-induced epidermal cells or fibroblast models, or streptozotocin-induced mouse models of DWs were not established for validation. Future studies should focus on exploring the effects of SDSS in these models. Second, the downstream pathways of PI3K-AKT (*e.g.*, mTOR) were not validated. Third, the use of LY294002 and OSI-0774 inhibitors alone may pose potential off-target effects. Future studies should employ more specific inhibitors or validate findings through gene knockdown or knockout techniques to establish more direct causal evidence. Lastly, although this study mainly revealed the crucial role of the EGFR-mediated PI3K-AKT pathway, it is also necessary to explore the molecular interaction network between SDSS and other high-priority targets (such as ESR1 and SRC) and further analyze the cross-regulatory mechanisms between EGFR and other signaling pathways (such as the calcium signaling pathway, *etc.*) beyond PI3K-AKT, MAPK, and JAK-STAT that were enriched, based on KEGG analysis. This will help deepen our understanding of the potential synergistic pathogenic pathways.

## 5 Conclusions

Using a multiomics strategy combining network pharmacology, machine learning, molecular docking, molecular dynamics simulation, and *in vitro* experiments, our study confirmed that SDSS could promote healing of DWs through multiple pathways and that SDSS activated the PI3K-AKT pathway in HUVECs by upregulating EGFR expression, thereby promoting endothelial cell migration and angiogenesis. SDSS simultaneously inhibited oxidative stress and inflammation, ultimately protecting cells from cellular damage in a high-glucose environment. Our findings collectively provide a theoretical basis for the clinical use of SDSS in treating DWs and serve as an important reference for the development of novel therapeutic drugs in the management of DWs.

## Author contributions

Peng Ning: data curation, formal analysis, investigation, methodology, visualization, writing – original draft. Fan Yang: funding acquisition, resources. Hongyi Cao: resources, supervision. Bo Zhou: conceptualization, supervision, writing – review & editing.

## Conflicts of interest

All authors state that they have no conflicts of interest regarding the publication of this paper.

## Supplementary Material

RA-016-D5RA09417H-s001

RA-016-D5RA09417H-s002

RA-016-D5RA09417H-s003

## Data Availability

The data supporting the findings of this study are available from the corresponding author upon reasonable request. All relevant data generated or analyzed during this study are included in this published article. Supplementary information (SI): SI1: hyperparameter configuration, training-testing performance comparison, 10-fold cross-validation results, volumes of grid boxes binding energies, and raw figures. SI2: machine learning code. SI3: complete target data. See DOI: https://doi.org/10.1039/d5ra09417h.
