# Supplementary material for: Sodium Danshensu promotes diabetic wound healing by targeting the EGFR-mediated PI3K-AKT pathway: a combined network pharmacology, machine learning, and in vitro approach
Source: RSC Adv. 2026 Mar 10;16(15):13394–407. doi: 10.1039/d5ra09417h (PMC12972934; doi:10.1039/d5ra09417h)
Supplement: RA-016-D5RA09417H-s001 [file RA-016-D5RA09417H-s001.pdf]

Supplementary Information (SI) for RSC Advances.
This journal is © The Royal Society of Chemistry 2026

Supplementary Material 1

Sodium Danshensu promotes diabetic wounds healing by targeting the EGFR-mediated PI3K-AKT pathway: A combined network pharmacology, machine learning, and *in vitro* approach

Peng Ning et al.,

● Supplementary Data

Data S1. Complete hyperparameter configuration in machine learning.

Data S2. Training-testing performance comparison in machine learning.

Data S3. 10-fold cross-validation results in machine learning.

Data S4. The volume of the grid boxes with the 6 lowest binding energies in molecular docking.

● Supplementary Figure

Figure S1. Raw figure of EGFR and GAPDH amplification and melting curves of qRT-PCR.

Figure S2. Raw figure from the Western blot in Fig. 8B.

Figure S3. Raw figure from the Western blot in Fig. 8C.

Figure S4. Raw figure from the Western blot in Fig. 8D.

```
{
  "timestamp": "2026-01-26 19:56:34",
  "dataset_size": 121,
  "n_features": 13,
  "training_size": 84,
  "test_size": 37,
  "classification_threshold": 0.26897241721977094,
  "best_rf_classifier_params": {
    "max_depth": 10,
    "min_samples_leaf": 1,
    "min_samples_split": 2,
    "n_estimators": 100
  },
  "best_rf_classifier_cv_score": 0.9595959595959596,
  "best_xgb_classifier_params": {
    "colsample_bytree": 0.8,
    "learning_rate": 0.01,
    "max_depth": 3,
    "n_estimators": 100,
    "subsample": 0.8
  },
  "best_xgb_classifier_cv_score": 0.9777777777777779,
  "best_rf_regressor_params": {
    "max_depth": 10,
    "min_samples_leaf": 1,
    "min_samples_split": 2,
    "n_estimators": 100
  },
  "best_rf_regressor_cv_score": 0.9779085261713109,
  "best_xgb_regressor_params": {
    "colsample_bytree": 0.8,
    "learning_rate": 0.1,
    "max_depth": 3,
    "n_estimators": 200,
    "subsample": 0.8
  },
  "best_xgb_regressor_cv_score": 0.957092982765905
}
```

Data S1. Complete hyperparameter configuration in machine learning.

Model	Train Accuracy	Test Accuracy	Accuracy Diff	Train F1	Test F1	F1 Diff
Random Forest	1.000	0.973	0.027	1.000	0.952	0.048
XGBoost	0.988	0.973	0.015	0.980	0.952	0.028

Data S2. Training-testing performance comparison in machine learning.

Random Forest	XGBoost
1.0	1.0
0.8	1.0
1.0	1.0
1.0	1.0
0.8	0.8
1.0	1.0
1.0	1.0
1.0	1.0
1.0	1.0
1.0	1.0

Data S3. 10-fold cross-validation results in machine learning.

PPARG	EGFR	ESR2
center_x = 18.3<br>center_y = -3.3<br>center_z = 13.8<br><br>size_x = 24.3<br>size_y = 57.5<br>size_z = 22.9<br><br>num_modes = 1	center_x = -13.3<br>center_y = 14.0<br>center_z = -25.6<br><br>size_x = 21.3<br>size_y = 22.1<br>size_z = 22.3<br><br>num_modes = 1	center_x = 22.4<br>center_y = 8.2<br>center_z = 113.5<br><br>size_x = 20.4<br>size_y = 13.7<br>size_z = 18.8<br><br>num_modes = 1

ESR1	IGF1R	CASP3
center_x = -7.4<br>center_y = -4.4<br>center_z = 12.8<br><br>size_x = 17.3<br>size_y = 14.3<br>size_z = 20.6<br><br>num_modes = 1	center_x = 6.3<br>center_y = -7.7<br>center_z = 21.6<br><br>size_x = 17.0<br>size_y = 23.1<br>size_z = 17.5<br><br>num_modes = 1	center_x = 30.1<br>center_y = 24.0<br>center_z = 40.1<br><br>size_x = 65.6<br>size_y = 58.6<br>size_z = 57.0<br><br>num_modes = 1

Data S4. The volume of the grid boxes with the 6 lowest binding energies in molecular docking.

Amplification Curve

EGFR

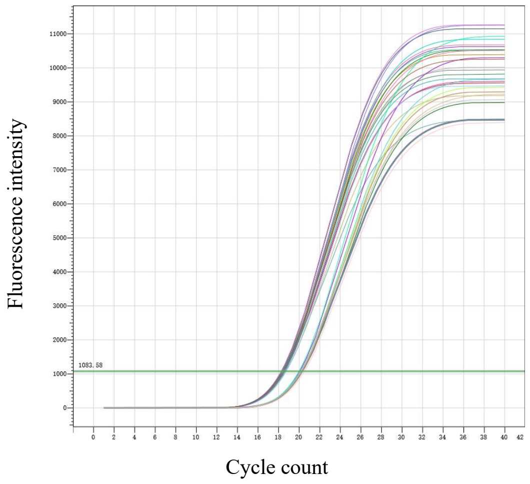

GAPDH

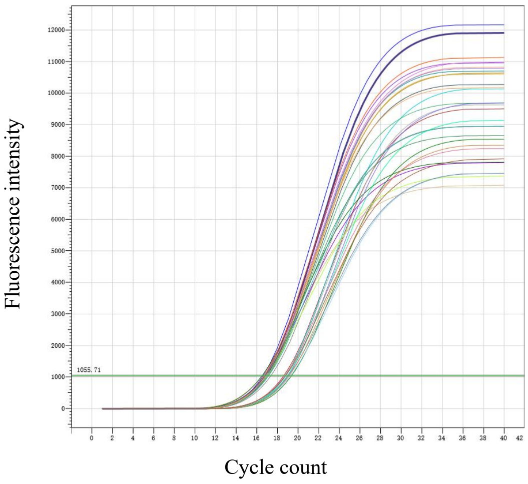

Melting Curve

EGFR

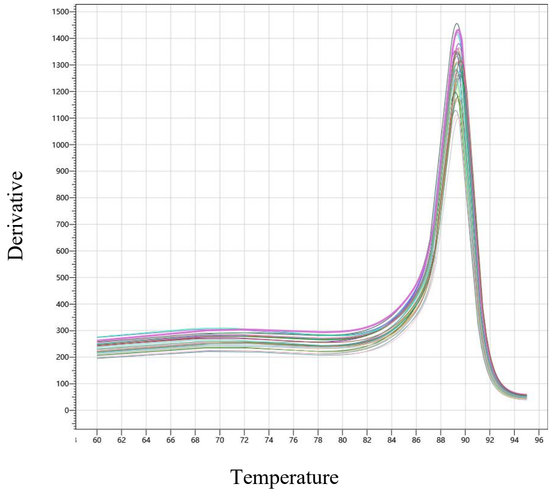

GAPDH

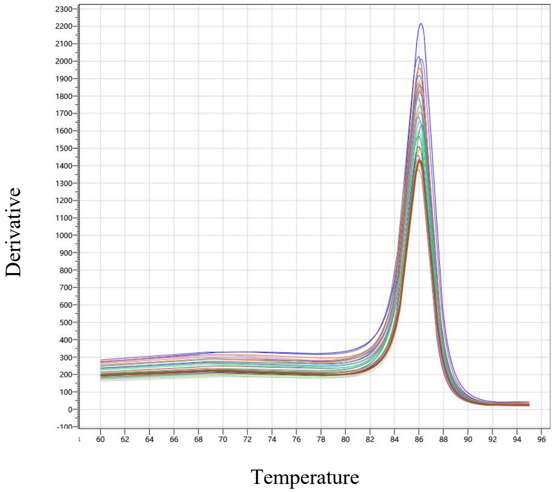

Figure S1 Raw figure of EGFR and GAPDH amplification and melting curves of qRT-PCR.

Fig. 8B

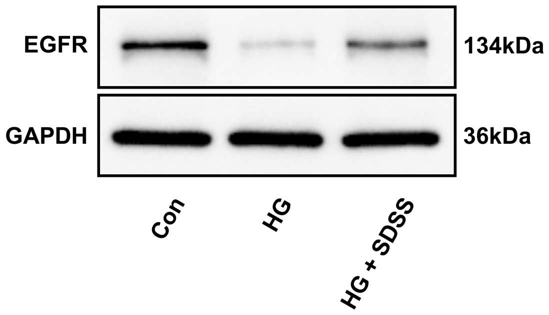

EGFR

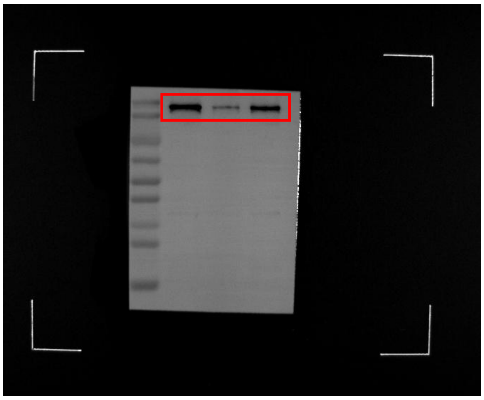

GAPDH

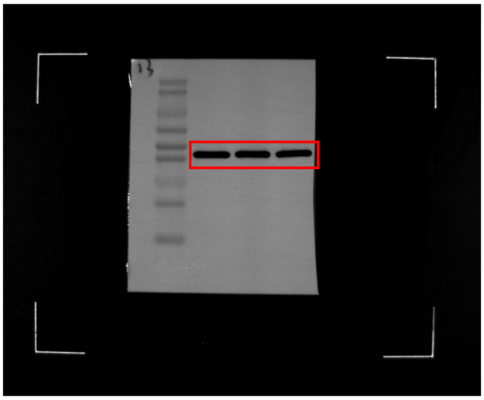

Figure S2. Raw data from the Western blot in Fig. 8B.

Fig. 8C

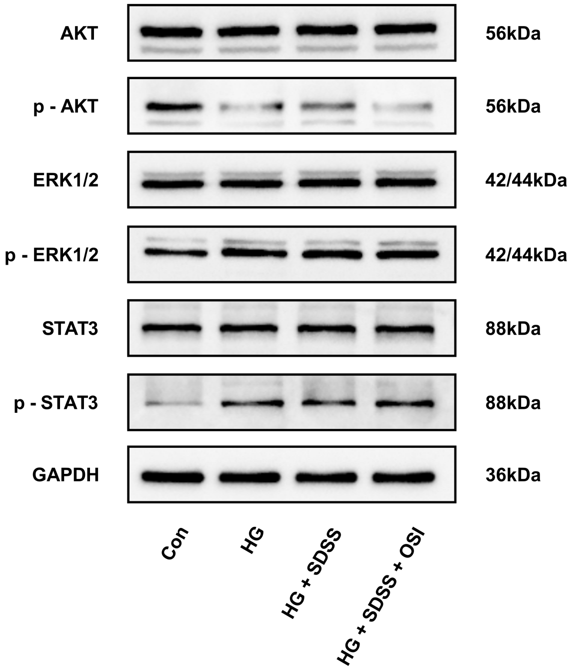

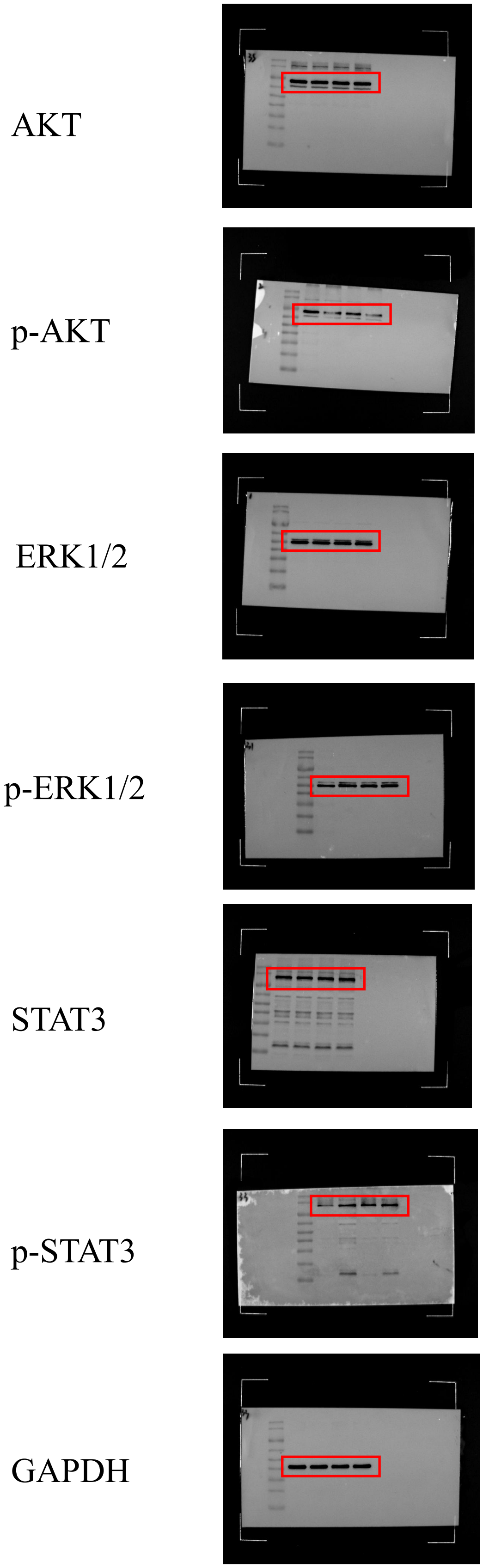

Figure S3. Raw figure from the Western blot in Fig. 8C.

Fig. 8D

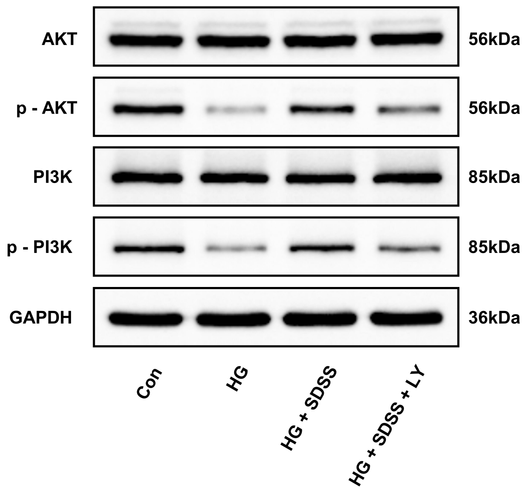

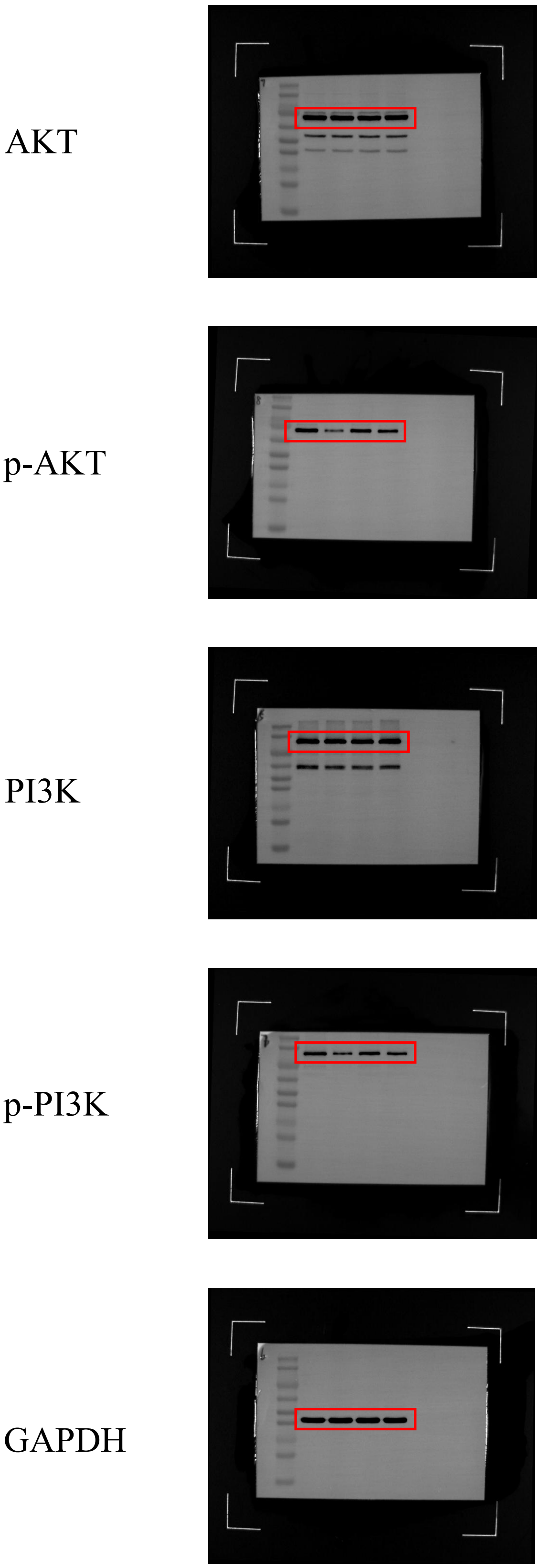

Figure S3. Raw figure from the Western blot in Fig. 8D.
